# The association between disability and physical performance, pain intensity, and pain-related anxiety in patients after lumbar decompression surgery: a cross-sectional study

**DOI:** 10.1186/s13018-023-04462-5

**Published:** 2023-12-13

**Authors:** Mohamad Sahebalam, Shabnam ShahAli, Khalil Komlakh, Sanaz Shanbehzadeh

**Affiliations:** 1 Iranian Center of Excellence in Physiotherapy, Rehabilitation Research Center, Department of Physiotherapy, School of Rehabilitation Sciences, Iran University of Medical Sciences, Tehran, Iran; 2 Imam Hossein Hospital, School of Medicine, Shahid Beheshti University of Medical Sciences, Tehran, Iran

**Keywords:** Disability, Decompression, Lumbar stenosis, Pain-related anxiety, Physical performance

## Abstract

**Background:**

Most patients with lumbar spinal stenosis improve significantly within 6 months of lumbar decompression surgery, however, unfavorable long-term disability may persist in some patients. It was unclear which potential influencing factors were more likely to be associated with disability. This study aimed to assess the association between disability and physical performance, pain, and pain-related anxiety in patients after lumbar decompression surgery.

**Methods:**

Patients who underwent decompression for lumbar spinal stenosis were included. Participants completed the visual analog scale, Oswestry Disability Index, and Pain Anxiety Symptoms Scale-20 to collect pain intensity, disability, and pain-related anxiety information. For physical performance assessment, participants performed timed up and go (TUG), functional reach test (FRT), 6-min walking test, and modified Sorensen test, 6–12 months after lumbar decompression surgery. The associations were examined with bivariate and multivariable linear regression analyses.

**Results:**

A total of 80 patients were included. A significant association between disability and pain-related anxiety, the FRT, and the modified Sorensen test scores was confirmed in multivariable analyses. Both bivariate (*r* = − 0.75) and multivariable (*β* = 0.60, 95% CI, 0.24, 0.54; *P* = 0.00) analyses confirmed that pain-related anxiety was the strongest indicator of disability. The association between disability and pain intensity, TUG, and 6-min walking test scores was not confirmed.

**Conclusion:**

Pain-related anxiety should be considered in the rehabilitation programs after lumbar decompression surgery. The evaluation of all aspects of physical performance following lumbar decompression surgery is also recommended.

## Introduction

Lumbar spinal stenosis is a common source of low back pain (LBP). It refers to a central canal narrowing or foraminal stenosis leading to leg and back pain. If conservative management of lumbar spinal stenosis is unsuccessful, surgical decompression surgery may be advocated to relieve symptoms [1]. Although most patients with lumbar spinal stenosis improve markedly within 6 months following lumbar decompression surgery [2], an unfavorable long-term disability remains in some patients [3].

Lumbar spinal stenosis is an important cause of disability [1]. Disability is a core issue in LBP, affecting physical performance and aerobic fitness [4]. Many factors such as psychosocial factors, pain intensity, and functional capacity, are reported to be related to disability in LBP individuals [5–7]. Psychological factors such as high levels of pain-related anxiety are recognized as a predisposing factor for avoiding physical activity, which leads to disability development and pain maintenance [5].

Physical performance tests are used to assess the functional capacity of people with chronic pain and in particular LBP [8]. The objective physical performance tests use standardized methodology and provide a more comprehensive measure of a person’s function than self-reported measures of physical performance [8]. The 6-min walking test (6MWT), functional reach test (FRT), timed up and go (TUG), and modified Sorensen test are used in clinical practice to evaluate the different aspects of physical performance in individuals with LBP [9–12].

It is unclear which of the potentially influencing factors (physical performance, pain, and pain-related anxiety) could be a stronger indicator of disability in patients after lumbar decompression surgery. Understanding the association of disability with physical performance, pain, and pain-related anxiety, can be helpful in the development of interventions aimed at modifying these determinants in patients after lumbar decompression surgery.

The purpose of this study was to evaluate the association between disability and physical performance, pain, and pain-related anxiety in patients after lumbar decompression surgery.

## Methods

### Study design

An observational cross-sectional study was performed from September 2021 to September 2022 in Tehran, Iran. The study was approved by the Ethics Committee of Iran University of Medical Sciences (IR.IUMS.REC.1398.806). All participants signed informed consent before enrollment in the study.

### Participants

Participants were recruited via convenience sampling from one university hospital in Tehran, Iran. Adult patients that underwent lumbar decompression surgery without fusion for spinal stenosis by a single surgeon, were included. Evaluation of disability, pain intensity, pain-related anxiety, and physical performance was performed 6–12 months following surgical treatment.

The inclusion criteria were: adults aged 18–60 years, receiving primary lumbar decompression surgery without fusion, and being able to actively participate in the program. Exclusion criteria were: history of prior thoracic, lumbar, or lower extremity surgery, a body mass index (BMI) greater than 35, other neurological diseases, Cobb angle of > 30, pregnancy, decompression for trauma or malignancy, and more than 2-level operation.

### Measures

#### Independent variables

##### Physical performance

Functional mobility was assessed using the TUG test. TUG has demonstrated acceptable reliability and validity in subjects after surgery for lumbar degenerative diseases [10]. To perform the test, participants were instructed to stand up from a chair, walk 3 m at their comfortable speed, turn, walk back toward the chair, and sit down. The duration of the test was measured in seconds using a chronometer [10].

Functional exercise capacity was measured using the 6MWT. 6MWT is a reliable and valid tool for the assessment of functional exercise capacity in chronic pain trials [9]. The test measures the distance that an individual can walk on a flat surface over a total of six minutes. The more distance traveled; the better performance is recorded [9].

Dynamic balance was assessed using the FRT. Participants were asked to stand next to the wall while keeping their arms straight at shoulder level and reach forward as far as possible, without stepping. The distance between the length of the arm and a maximal forward reach was recorded in the standing position. Longer reaching distances indicate better dynamic balance [11].

Back muscle endurance was evaluated by the modified Sorensen test which is the most widely used test in the literature for evaluating the isometric endurance of back extensor muscle. Participants were positioned prone on an examination table with the upper edges of their iliac crests aligned with the table’s edge. Both lower extremities were fixed to the examining table using three straps at the levels of the pelvis, knees, and ankles. With their arms folded across their chest, participants were instructed to hold their trunks at a horizontal position relative to the ground as long as possible. The time that the participant was able to hold the test position was measured in seconds. Maximum holding time was considered as endurance performance [12].

##### Pain intensity

Pain intensity was assessed using the visual analog scale (VAS). The scale is a self-administered measure, ranging from 0 points which indicates "no pain" to 10 points which indicates "maximum intensity of pain." The participants rated their current pain intensity using VAS [13].

##### Pain- related anxiety

Pain-related anxiety was assessed via the Persian short-form version of the Pain Anxiety Symptoms Scale-20 (PASS-20) [14]. The scale is a self-administered questionnaire with 20 items that are rated on a 6-point Likert scale (0 = never, 5 = always). Total score ranges from 0 to 100, with higher scores indicating greater pain-related anxiety. The tool indicates good reliability and validity in LBP subjects [14].

### Dependent variable

#### Disability

Disability was evaluated by the Oswestry Disability Index (ODI). The ODI is a self-administered, 10-item questionnaire designed to assess pain-related disability in individuals with LBP. Each item is scored on a 0–5 scale, with 0 demonstrating no disability and 5 demonstrating the greatest disability. Total score ranged between 0 to 100 percent and higher scores mean higher disability. The Persian version of ODI has shown reliability and validity properties in LBP subjects [15]. According to the cut-off score of 22, participants were considered as of low and high disability [16].

#### Procedures

Age, sex, height, and weight were recorded at baseline. All participants completed the Persian version of the VAS, ODI, and PASS-20, to collect pain intensity, disability, and pain-related anxiety information. For physical performance assessment, participants perform TUG, FRT, 6MWT, and modified Sorensen test at the same session in a random order. A five-minute rest time was considered between each test to minimize fatigue. All tests were performed by one experienced physiotherapist.

### Statistical analysis

The sample size was calculated with G*Power, version 3.1.9.2 for a linear multiple regression model. From a priori analysis, with a power of 80%, *α* = 0.05, the effect size of *f* = 2.5, and the total number of predictors and covariates as 8, the required sample size would be 69. Considering 15% dropout, 80 patients were included.

Data analysis was performed using SPSS software, version 26. Numerical data was expressed as mean and standard deviation, while categorical data was presented as frequency and percentage. The normality of the data was verified using a combination of histograms, the Shapiro‐Wilks test, and skew/kurtosis. The independent t-test was used to compare study variables between groups with high and low disability.

To evaluate the bivariate correlation between physical performance, pain-related anxiety, and disability, Pearson’s correlation was used.

A multivariable linear regression analysis was performed to evaluate the contribution of dependent variables (physical performance, pain-related anxiety, and pain intensity) with the independent variable (disability) in patients after lumbar decompression surgery. To control for demographic variables (age, gender, BMI) that could be associated with disability, these variables were selected priori as potential covariates. Only variables with significant bivariate correlation were included in the model. Before performing linear regression analysis, the required assumptions were tested: linearity (scatterplots), normality of dependent variable, and homoscedasticity were tested by plotting the residuals versus the fitted values, and the presence of multicollinearity was determined by a variance inflation factor > 3. The correlation between all independent variables used in the regression analysis was < 0.80. All variables with significant correlations with ODI score were included in a multivariable linear regression analysis. Standardized β values are presented to reflect the direction and strength of the association between each independent variable.

Finally, a stepwise regression analysis was performed with the disability as the dependent variable and only the significant independent variables from the preliminary multivariable analysis. The level of statistical significance was set at *p* < 0.05.

## Results

Eighty individuals who underwent lumbar decompression surgery were included. According to the normality tests, the distribution of data was normal. Table 1 illustrates the comparison of demographic data and functional tests between high and low-disability individuals. The results of the independent *t*-test showed that there was no significant difference in age and BMI between groups. Individuals with high disability showed significantly higher TUG test time, and lower FRT scores compared to low disability individuals. Moreover, individuals with a higher disability had lower modified Sorensen test times as well. No significant between-group differences were observed for 6MWT scores. In addition, higher PASS-20 scores and greater pain intensity in both low back and lower extremity regions were observed in high-disability individuals.

**Table 1:** Demographic information and clinical characteristics of participants in high (*n* = 40) and low disability (*n* = 40) groups

Variable	Group	Mean (SD)	Number (Percent)	P value
Age (year)	High disability	45.05 (9.54)	–	0.11
	Low disability	41.55 (9.98)	–	
BMI (Kg/m^2^)	High disability	25.19 (3.21)	–	0.21
	Low disability	24.35 (2.85)	–	
Gender (Female/ Male)	High disability	–	22 (55%)/ 18 (45%)	-
	Low disability	–	16 (40%)/ 24 (60%)	
ODI (0%-100%)	High disability	37.48 (8.18)	40 (50%)	0.00
	Low disability	14.23 (5.08)	40 (50%)	
6MWT(m)	High disability	319.77 (44.33)	–	0.66
	Low disability	407.76 (295.24)	–	
TUG (sec)	High disability	10.27 (1.70)	–	0.00
	Low disability	8.37 (1.70)	–	
FRT (cm)	High disability	25.35 (5.47)	–	0.00
	Low disability	33.43 (4.98)	–	
Modified Sorensen test (sec)	High disability	10.75 (18.41)	–	0.00
	Low disability	65.96 (31.79)	–	
VAS (back pain) (cm)	High disability	2.38 (1.86)	–	0.00
	Low disability	0.63 (1.06)	–	
VAS (lower extremity pain) (cm)	High disability	2.67 (1.68)	–	0.00
	Low disability	0.68 (1.45)	–	
PASS-20 (0–100 points)	High disability	47.63 (18.04)	–	0.00
	Low disability	16.75 (7.84)	–	
Time since post-surgery (month)	High disability	9.27 (2.08)	–	0.09
	Low disability	8.95 (2.41)	–	

*BMI* body mass index, *FRT* functional reach test, *ODI* Oswestry Disability Index, *PASS-20* Pain Anxiety Symptoms Scale-20, *TUG* timed up and go test, *VAS* visual analog scale, *6MWT* 6-min walking test

### Bivariate association

The results of bivariate association revealed a significant relationship between physical performance (FRT and modified Sorenson), VAS, and PASS-20 with disability, except 6MWT which showed no significant association. In addition, the strongest association was observed between disability and PASS-20 (*r* = − 0.75). Table 2 also shows the correlation among study variables. PASS-20 showed a strong negative relationship with FRT and modified Sorenson and a moderate positive association with VAS of leg and back pain.

**Table 2:** Bivariate correlation analysis (*n* = 80)

Variable	1	2	3	4	5	6	7	8
1. ODI	1							
2. 6MWT	− 0.19	1						
3. TUG	0.41**	0.24*	1					
4. FRT	− 0.53**	0.29**	− 0.69**	1				
5. Modified Sorensen test	− 0.71**	0.34**	− 0.66**	0.75**	1			
6. VAS (back pain)	0.50**	− 0.12	0.36**	− 0.38**	− 0.50**	1		
7. VAS (Lower extremity pain)	0.55**	− 0.10	0.44**	− 0.50**	− 0.53**	0.68**	1	
8. PASS-20	0.75**	− 0.19	0.49**	− 0.74**	− 0.70**	0.51**	0.62**	1

*FRT* functional reach test, *ODI* Oswestry Disability Index, *PASS-20* Pain Anxiety Symptoms Scale-20, *TUG* timed up and go test, *VAS* visual analog scale, *6MWT* 6-min walking test

*P < 0 .05

**P < 0.01

### Linear regression analysis

All tested assumptions met the criteria for conducting a linear regression analysis. Table 3 demonstrates the results of the multivariable linear regression analysis. The results of the preliminary multivariable linear regression analysis revealed a significant contribution of PASS-20, modified Sorensen test, and FRT, to ODI (disability); therefore, these variables were included in the second multivariable analysis. A significant overall model emerged (*F* = 19.25, *P* < 0.001), explaining around 68% of ODI variance. Among these predictors, PASS-20 was the strongest predictor of ODI.

**Table 3:** Multivariable linear regression analysis, representing the relation between dependent variables (physical performance, psychological, and pain intensity) with the independent variable (disability) (*n* = 80)

Dependent variables or covariates	Disability (ODI)	
	*β* (95% CI)	*P* value
Age (year)	0.07 (− 0.14, 0.34)	0.40
BMI (Kg/m2)	− 0.08 (− 1.02, 0.28)	0.26
Gender (Female^(R)^)	− 0.04 (− 5.59, 2.93)	0.53
TUG (sec)	− .091(− 2.19, 0.93)	0.42
FRT (cm)	0.27 (0.04, 1.07)	0.03*
Modified Sorensen test (sec)	− 0.50 (− 0.26, − 0.09)	0.00*
VAS (back pain) (cm)	0.07 (− 0.68, 1.81)	0.36
PASS-20 (0–100 points)	0.60 (0.24, 0.54)	0.00*
F	19.25	
R	0.833	
R2 (%)	68.4	

*P < 0.05

*BMI* body mass index, *FRT* functional reach test, *ODI* Oswestry Disability Index, *PASS-20* Pain Anxiety Symptoms Scale-20, *TUG* timed up and go test, *VAS* visual analog scale

Only the significant independent variables used in the multivariable regression analysis were included in the stepwise regression. Stepwise regression analysis (Table 4) revealed that PASS-20 (model 1) accounted for 57% of the variance score on the ODI. In addition, Sorenson and FRT, (model 3) explained up to 67% of the variance in the level of ODI (disability).

**Table 4:** Stepwise linear regression analysis

Model	Predictor	*R*	*R*^2^	*P* value
1	PASS-20	0.755	57%	> .0001
2	PASS-20 and Modified Sorensen test	0.799	64%	> .0001
3	PASS-20, Modified Sorensen test, and FRT	0.820	67%	> .0001

*FRT* functional reach test, *PASS-20* Pain Anxiety Symptoms Scale-20

## Discussion

This study aimed to describe the association between disability and physical performance, pain intensity, and pain-related anxiety among patients 6–12 months post-lumbar decompression surgery. The results revealed significant relationships between disability and pain-related anxiety, the FRT, and the modified Sorensen test scores in patients after lumbar decompression surgery. Pain-related anxiety was the strongest indicator of disability.

The findings showed that high levels of pain-related anxiety had the strongest association with disability. However, the level of pain intensity was not an indicator of disability. This might explain the importance of psychological factors as an indicator of long-term disability post-lumbar surgery. This finding is consistent with previous studies that reported a cumulative negative effect of different elevated psychosocial factors (e.g., fear of movement, catastrophizing, and depression) on long-term disability [17, 18]. Moreover, prior research suggests that pain-related anxiety is associated with disability in individuals with chronic non-specific LBP [19]. Individuals with chronic pain often experience negative emotions related to pain, as well as anxiety and worry about its negative consequences. Therefore, the individual may be more likely to avoid such aversive states by avoiding activities to cope with persistent pain and negative consequences [19].

Although the results of bivariate correlation support the association between disability and pain intensity, this association was not confirmed when other variables were entered into multivariable analyses. This finding demonstrates that high levels of pain-related anxiety, back muscle endurance (assessed by the modified Sorensen test), and dynamic balance (assessed by FRT) were more likely to be associated with disability. An assessment 6–12 months after lumbar decompression surgery may explain the lack of association between pain and disability. This may reflect that patients are experiencing symptom improvement, 6–12 months post-lumbar decompression surgery.

Disability was found to be associated with back muscle endurance (assessed by the modified Sorensen test) and dynamic balance (assessed by FRT). Also, the results of bivariate analysis revealed a strong positive association between modified Sorensen test scores and FRT scores in patients' post-lumbar decompression surgery. Performance of the Sorensen test and FRT involves trunk control and depends on back muscle endurance and strength [12, 20]. In patients who undergo lumbar decompression surgery, preoperative physical deconditioning and prolonged periods of inactivity after surgery may lead to the weakening of the back lumbar muscles [21]. Moreover, lumbar surgery can cause muscle damage and denervation, resulting in postoperative muscle atrophy [22]. Resection of the paraspinal muscles as well as changes in the paraspinal muscles' proprioception may affect trunk muscle strength and result in poor trunk control ability and postural instability [23]. A previous study found that balance improved 6–12 months after lumbar decompression surgery compared to pre-surgery. However, balance impairment was still observed compared to healthy individuals [24]. Dynamic balance control is crucial for performing upright standing tasks. Nevertheless, unstable balance control after surgery may increase the fall risk during activities of daily living [24]. Therefore, dynamic balance assessments and postoperative rehabilitation training are necessary after lumbar decompression surgery.

The results also revealed that while modified Sorensen test and FRT scores were strongly correlated with disability, TUG and 6MWT scores had no statistically significant correlation with disability. This finding aligns with previous research, which identified TUG test scores after lumbar decompression surgery was within the normal population range [25]. Modified Sorensen test and FRT examine spine-related functions and their performance depends on the endurance and strength of the back extensor muscles [12, 20] which are probably affected by surgery, whereas TUG and 6MWT provide global information regarding overall physical performance [26]. This finding highlights the importance of a comprehensive evaluation of all aspects of physical performance in patients after lumbar decompression surgery.

The results confirm that pain-related anxiety has the potential to improve disability in patients after lumbar decompression surgery; however, there is a need for additional targets to also improve back muscle endurance and dynamic balance, which are important aspects of physical performance.

### Limitations

The present study has some limitations. First, despite using valid and reliable questionnaires, the self-reported nature of the questionnaires might have led to recall bias. Second, the association of other psychological factors with disability (such as kinesiophobia, fear avoidance, …) was not assessed. Finally, the cross-sectional design of the study limits the evidence level of the findings.

## Conclusion

The presence of an association between disability and pain-related anxiety, and some aspects of physical performance (back muscle endurance, and dynamic balance) was supported in patients 6–12 months after lumbar decompression surgery. However, the association between disability and pain intensity, and some aspects of physical performance (functional mobility, and functional exercise capacity) could not be confirmed.

The results suggest that pain-related anxiety should be considered in the rehabilitation programs of patients after lumbar decompression surgery. Also, exercise programs that focus on back muscle endurance and dynamic balance may be crucial after lumbar decompression surgery. Moreover, a comprehensive evaluation of all aspects of physical performance is suggested in patients after lumbar decompression surgery.

## Data Availability

The data presented in this study are available on reasonable request from the corresponding author.

## Abbreviations

6MWT: 6-Minute walking test
BMI: Body mass index
FRT: Functional reach test
LBP: Low back pain
ODI: Oswestry Disability Index
PASS-20: Pain Anxiety Symptoms Scale-20
TUG: Timed up and go
VAS: Visual analog scale
